# Artemisinin protects against cerebral ischemia and reperfusion injury via inhibiting the NF-κB pathway

**DOI:** 10.1515/med-2022-0435

**Published:** 2022-05-11

**Authors:** Hui Ji, Haifeng Jin, Guangwei Li, Li Jin, Xiaoxu Ren, Ying Lv, Yuchun Wang

**Affiliations:** Department of Basic Medicine, Qiqihar Medical University, Qiqihar, Heilongjiang 161006, China; College of Pharmacy, Qiqihar Medical University, Qiqihar, Heilongjiang 161006, China

**Keywords:** cerebral infaction, artemisinin, NF-κB, inflammation, apoptosis, oxidative stress

## Abstract

This study investigated whether artemisinin (ART) exerts a neuroprotective effect against cerebral ischemia/reperfusion (I/R) injury. Hypoxia-glucose deprivation and reoxygenation (OGD/R) of SH-SY5Y cells were used as the I/R injury model *in vitro*. Cell viability was determined using 3-(4,5-dimethylthiazol-2-yl)-2,5-diphenyltetrazolium bromide assay, and lactate dehydrogenase (LDH) release was measured. Cell apoptosis and apoptosis-associated protein expression were determined via flow cytometry and western blotting, respectively. The levels of glutathione peroxidase, superoxide dismutase, catalase, and malondialdehyde were determined. The secretion of tumor necrosis factor-α and interleukin-1β was measured using ELISA. The activation of the nuclear factor kappa B (NF-κB) pathway was also determined. The indicated ART concentrations (0, 25, 50, 75, and 100 μM) had no significant effect on SH-SY5Y cell viability and LDH activity. ART promoted cell viability, reduced cell apoptosis, repressed cellular inflammation, and inhibited cellular oxidative stress and NF-κB signaling pathway in OGD/R-induced SH-SY5Y cells. In addition, all the protective effects of ART on OGD/R-induced SH-SY5Y cell injury were significantly reversed by an NF-κB agonist. In conclusion, ART protects neurons from OGD/R-induced damage *in vitro* by inhibiting the NF-κB signaling pathway. These results suggest that ART may be a potential agent for the treatment of cerebral I/R injury.

## 1 Introduction

*Artemisia annua* L. has been used as a medicinal plant in the treatment of numerous diseases for centuries [1]. Artemisinin (ART) is a sesquiterpene trioxane lactone compound extracted from *Artemisia annu*a L., which contains a peroxide group. ART can kill malaria parasites by interfering with mitochondrial function, particularly in the treatment of drug-resistant and recurrent malaria, and has been used worldwide [2,3]. In addition to anti-malarial effect, ART has immunosuppressive [4], anti-schistosomiasis [5], anti-virus [6], and anti-tumor effects [7,8,9,10]. In multiple cancers, combined treatment with ART drugs has achieved improved therapeutic effects [11], and no evident cytotoxicity of ART to normal cells was observed in more than 4,000 cases, which makes it a potential drug for treating numerous diseases [12]. ART has been reported to be a potential drug for treating Alzheimer’s disease by protecting against amyloid beta damage [13]. Studies have revealed that ART is involved in regulating the expression of various nuclear factor kappa B (NF-κB) reporter genes [14,15]. ART inhibits endometrial cancer cell proliferation by disrupting the interaction between NF-κB and the CDK4 promoter and transcriptionally downregulates CDK4 expression [9]. Gu et al. indicated that ART could inhibit the NF-κB pathway by blocking IKBα phosphorylation, which results in reduced myocardial remodeling [16]. Wang et al. found that ART pretreatment effectively protected against myocardial ischemia/reperfusion (I/R) injury by inhibiting the activation of the NLRP3 inflammasome [17]. Besides, ART attenuated the oxidative damage of SH-SY5Y cells and hippocampal neurons induced by hydrogen peroxide (H_2_O_2_) by activating the AMPK signaling pathway, suggesting a neuroprotective effect of ART [18]. However, whether ART has a neuroprotective effect on cerebral I/R injury remains unclear.

Ischemic stroke remains the primary cause of disability and death worldwide [19]. The pathogenesis of cerebral ischemia is insufficient oxygen and glucose transport caused by tissue ischemia, which leads to irreversible neuronal damage or death [20]. Recanalization therapy, which supplements nutrients and oxygen and removes toxic metabolites, is currently the main treatment method of ischemic stroke [21,22]. However, the recovery of blood flow leads to occasional side effects [23]. In the process of cerebral I/R, various procedures related to nerve cell death are activated, such as necrosis, apoptosis, or autophagy [24,25], and apoptosis has been reported as a key event in cerebral ischemic brain injury [26]. Astragaloside IV protects against cerebral I/R injury by suppressing apoptosis [27]. The inhibition of oxidative stress and inflammation has been widely reported to alleviate cerebral I/R injury [28,29].

In this study, we hypothesized that ART plays a protective role in cerebral ischemia and reperfusion injury by inhibiting NF-κB pathway. Therefore, this study employed the SH-SY5Y cell OGD/R model to explore the effect of ART on cerebral I/R injury and analyze its potential molecular mechanism.

## 2 Material and methods

### 2.1 Cell culture and reagent

The human neuroblastoma cell line SH-SY5Y was obtained from the American Type Culture Collection (Rockville, MD, USA) and was cultured in Dulbecco’s Modified Eagle Medium (DMEM; Sigma-Aldrich, St. Louis, MO, USA) containing 10% (v/v) fetal bovine serum (Thermo Fisher Scientific, Waltham, MA, USA), 2% l-glutamine (v/v), 100 μg/mL streptomycin, and 100 U/mL penicillin (Gibco, Amarillo, TX, USA). The cells were cultured in a moist environment at 37°C and 5% CO_2_. ART was purchased from Merck (Darmstadt, Germany; cat. no. 361593).

### 2.2 Cell treatment

OGD/R-exposed model [30]: SH-SY5Y cells were seeded into 96-well plates, and then OGD experiments were performed when cell density reached approximately 80% confluence. SH-SY5Y cell culture medium was replaced with oxygen-glucose-free DMEM and incubated in an anaerobic gas mixture (1% O_2_, 5% CO_2_, and 94% N_2_) at 37°C. After 3 h, the cell medium and culture conditions were restored to normal, and the culture was continued for 24 h.

ART interferes with the OGD/R-exposed model: SH-SY5Y cells were pre-treated with the indicated concentration of ART (0, 25, 50, and 100 μM) [31] for 2 h and then exposed to OGD/R.

### 2.3 Cell proliferation ability

The proliferation ability of SH-SY5Y cells was determined using the MTT assay. The processed cells were seeded into 96-well plates at a density of 6 × 10^3^ cells per well and cultured in 5% CO_2_ at 37°C for 48 h. Subsequently, 20 μL of MTT solution (5 mg/mL) was added to each well and cultured for 4 h. Then, the supernatant was carefully removed, and 150 μL of dimethyl sulfoxide was added to each well at room temperature to completely dissolve the formazan crystals. The absorbance of each well was measured at 570 nm using an enzyme-linked immunosorbent analyzer (Victor X3, PerkinElmer, Shelton, CT, USA).

### 2.4 LDH release cell death assay

An LDH assay kit (Promega, Madison, WI, USA) was used to detect the release of LDH from cells. Briefly, 2 × 10^6^ processed cells were collected, washed with pre-cold phosphate-buffered saline (PBS), resuspended in 200 μL of cold assay buffer, and centrifuged at 4°C at 8,000 rpm for 15 min, and the supernatant was preserved. Next 10 μL of supernatant and 100 μL of reaction reagent were mixed and incubated for 30 min at room temperature. The absorbance of each well was determined using an enzyme-linked immunosorbent analyzer (Victor X3, PerkinElmer) at 490 nm.

### 2.5 Flow cytometry analysis of cell apoptosis

SH-SY5Y cell apoptosis was detected using the Annexin V-FITC Assay Kit (BioVision, Palo Alto, CA, USA) according to the manufacturer’s instructions. Briefly, the processed SH-SY5Y cells were collected and washed with pre-cold PBS. SH-SY5Y cells were resuspended in 195 μL of binding buffer, and then stained with 5 μL of Annexin V-FITC (10 μg/mL) and 10 μL of propidium iodide for 15 min in the dark at 25°C. Finally, the cells were analyzed via flow cytometry (FCM; Beckman, Brea, CA, USA). Data were analyzed using FlowJo version 7.6.1 (FlowJo LLC).

### 2.6 Caspase-3 activity detection

Caspase-3 activity was detected using a Caspase-3 Activity Assay Kit (Abcam, Cambridge, UK; cat no. ab252897). The detection principle is that the synthetic substrate DEVD-AFC will emit a strong and stable fluorescence signal (Ex/Em = 400/505 nm) after being cleaved by caspase-3, and caspase-3 enzyme activity can be reflected according to the fluorescence intensity. The processed cells were collected and lysed with RIPA lysis buffer (Merck; cat no. R0278), and the supernatant was collected. The supernatant was then incubated with the final reaction solution, which comprised 40 μL of assay buffer, 50 μL of supernatant, and 10 μL of caspase-3 substrate DEVD-AFC (2 mM) at 37°C for 2 h. Finally, the absorbance of each sample was measured at 400 nm using an enzyme-linked immunosorbent analyzer (Victor X3, PerkinElmer,).

### 2.7 Western blot analysis

SH-SY5Y cells were washed with PBS and lysed with RIPA lysis buffer (Merck; cat. no. R0278). The supernatant with 20 μg total protein content was separated via polyacrylamide gel electrophoresis and electro-transferred to a nitrocellulose membrane (Millipore, Burlington, MA, USA). The membranes were incubated with specific primary antibodies (1:200 for anti-cleaved caspase-3, cat. no. ab2302; 1:1,000 for anti-p65, cat. no. ab32536; 1:500 for anti-p-p65, cat. no. ab31624; 1:1,000 for anti-GAPDH, cat. no. ab22555; all from Abcam), and secondary antibodies (1:5,000, cat. no. ab97080; Abcam). The enhanced chemiluminescence method (Cytiva, Marlborough, MA, USA) was used to detect immune-response bands. The band intensity was quantified by ImageJ version 1.8.0 (National Institutes of Health).

### 2.8 Determination of superoxide dismutase (SOD), catalase (CAT), and glutathione peroxidase (GSH-Px) activities

Superoxide dismutase activity assay kit (Solarbio, Beijing, China; cat. no. BC0170) was used to determine the activity of SOD, catalase detection kit (Solarbio; cat. no. BC0200) was used to determine the activity of CAT, and glutathione peroxidase activity assay kit (Solarbio; cat. no. BC1190) was used to determine GSH-Px activity. In the SOD determination system, xanthine and xanthine oxidase reaction can produce O^2−^, which reduces nitroblue tetrazolium to formazan, and SOD reduces formazan formation by reacting with O^2−^. Formazan can be dissolved in organic solvents, and absorbance at 560 nm indicates the SOD activity. CAT can decompose H_2_O_2_, which has a characteristic absorption peak at 240 nm. The absorbance of the reaction solution at 240 nm decreased with the reaction time, and the CAT activity was calculated according to the change rate of the absorbance. GSH-Px catalyzes the oxidation of GSH by H_2_O_2_ to produce oxidized glutathione. GSH reacts with 5,5’-dithiobis-(2-nitrobenzoic acid) to generate a compound with a characteristic absorption at 412 nm. Therefore, the absorbance of the reaction solution at 412 nm was used to measure the activity of GSH-Px.

### 2.9 Malondialdehyde (MDA) detection

A Malondialdehyde Assay Kit (Abcam; cat. no. ab118970) was used to detect MDA. MDA in the sample reacts with thiobarbituric acid (TBA) to form an MDA–TBA complex. The MDA–TBA complex had a specific absorption at 532 nm. Therefore, MDA was quantified according to the absorbance of the reaction solution at 532 nm.

### 2.10 Enzyme-linked immunosorbent assay (ELISA)

The secretion of tumor necrosis factor-α (TNF-α), interleukin-1β (IL-1β), and IL-6 in SH-SY5Y cells was detected using an ELISA kit (cat. no. ab181421 for TNF-α; cat. no. ab214025 for IL-1β; cat. no. ab178013 for IL-6; all from Abcam). The cell culture supernatant was collected by centrifugation at 2,000 rpm for 15 min. Afterward, 50 μL of cell culture supernatant and 50 μL of antibody cocktail were added to the 96-well plate included in the kit, incubated in a shaker at 400 rpm for 90 min at room temperature, and then washed three times with 350 μL of wash buffer. Subsequently, 100 μL TMB buffer was added to the system and incubated in a shaker at 400 rpm for 10 min. After incubation, the reaction was stopped with 100 μL of stopping solution. Absorbance was measured at 450 nm wavelength.

### 2.11 Reverse transcription quantitative-PCR (RT-qPCR)

The p65 transcription level was determined via RT-qPCR. Total RNA was extracted using the MiniBEST Universal RNA Extraction Kit (TaKaRa, cat. no. 9767), according to the manufacturer’s instructions. A One Step PrimeScript III RT-qPCR kit (TaKaRa, cat. no. RR600A) was used for the RT-qPCR analysis. The reaction solution was prepared according to the manufacturer’s instructions and applied in a Thermal Cycler Dice Real Time System. GAPDH was used as an internal control. The PCR primer sequence of p65 and GAPDH was as follows: p65 forward, 5′-CGCGGATCCGCCACCATGGACGAACTG-3′ and reverse, 5′-CCGCTCGAGTTAGGAGCTGATCTG-3′; GAPDH forward, 5′-CTTTGGTATCGTGGAAGGACTC-3′ and reverse, 5′-GTAGAGGCAGGGATGATGTTCT-3′. The relative expression of p65 was calculated using the 2^−ΔΔCq^ method.

### 2.12 Statistical analysis

All experiments were performed at least three times, and the data are presented as mean value ± SD. GraphPad Prism software (GraphPad Software, Inc., San Diego, CA, USA) was used for the statistical analysis. The statistical significance of the differences between the two groups was tested using Student’s *t*-test. Multiple comparisons were performed using the one-way analysis of variance (ANOVA) followed by Tukey’s *post hoc* test. Differences were considered statistically significant at *p* < 0.05.

## 3 Results

### 3.1 Cytotoxicity of ART to SH-SY5Y cells

**Figure 1:**
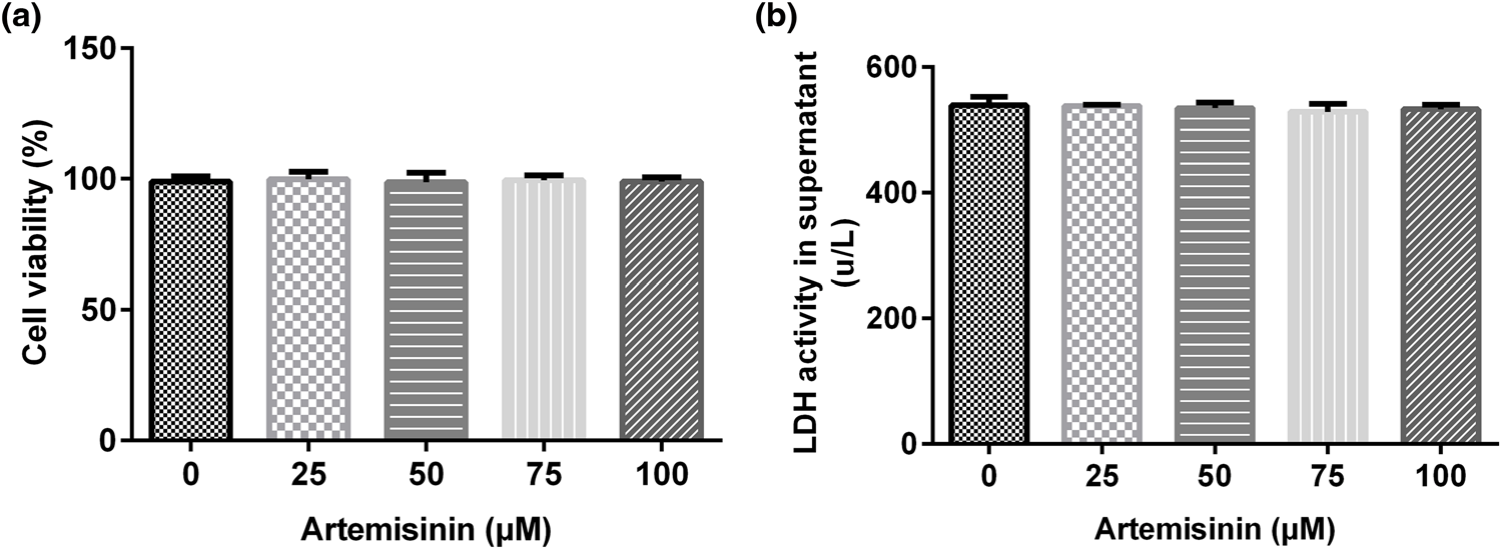
Cytotoxic effect of ART on SH-SY5Y cells. SH-SY5Y cells were treated with ART at different concentrations (0, 25, 50,75, and 100 μM) for 24 h. Different ART concentrations had no significant effect on SH-SY5Y: (a) cell viability and (b) lactate dehydrogenase activity.

SH-SY5Y cells were treated with ART at different concentrations (25, 50, 75, and 100 μM) for 24 h. Different ART concentrations had no significant effect on SH-SY5Y cell viability and LDH viability (Figure 1a and b), suggesting that ART has no significant cytotoxic effect on SH-SY5Y cells. Therefore, 0, 25, 50, and 100 μM ART were used for subsequent experiments.

#### 3.1.1 ART protects SH-SY5Y cells from OGD/R-induced injuries

**Figure 2:**
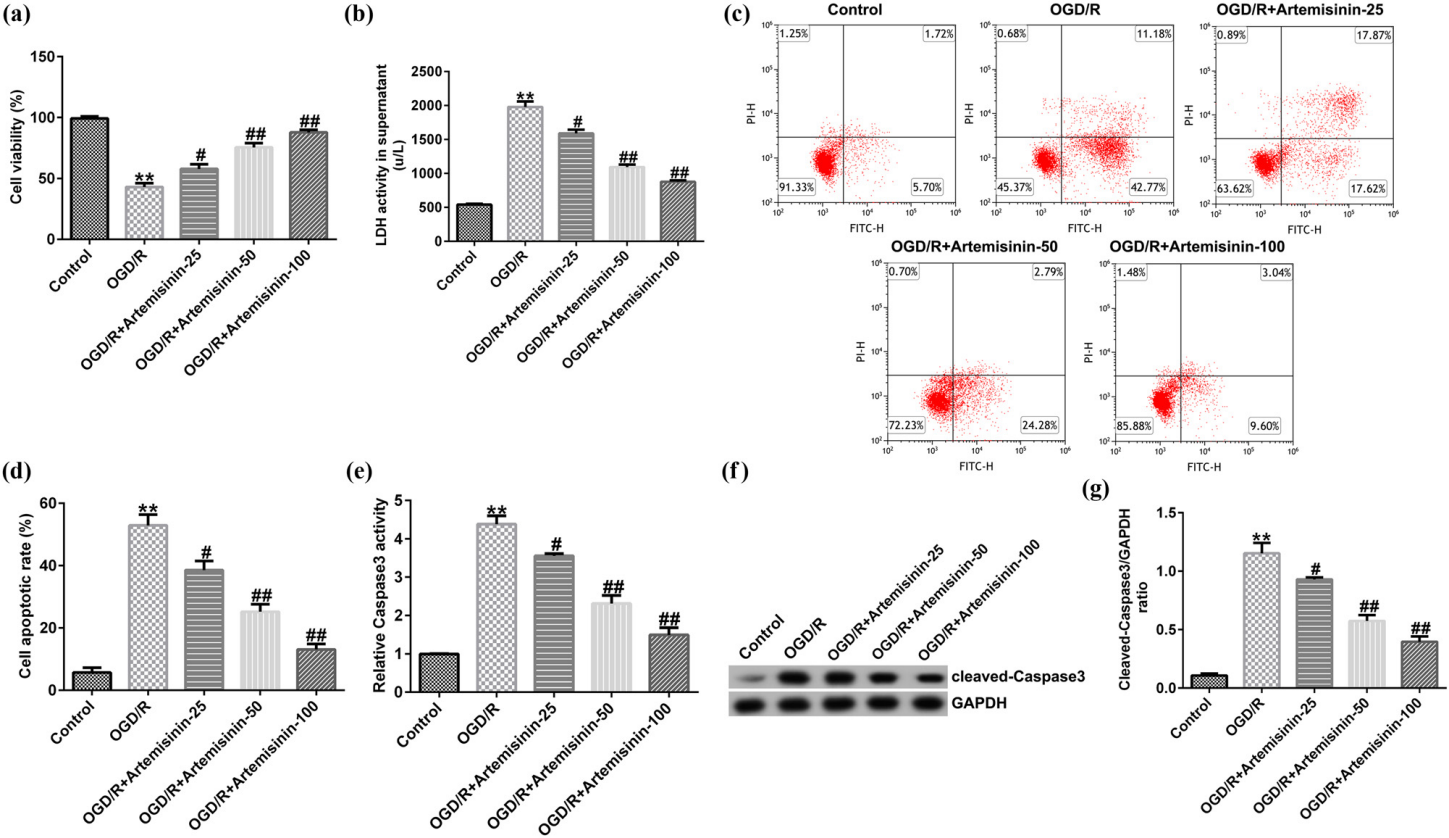
ART increases the viability and reduces apoptosis of hypoxia-glucose deprivation and reoxygenation (OGD/R)-exposed SH-SY5Y cells in a dose-dependent manner. SH-SY5Y cells were pre-treated with the indicated concentrations of ART (0, 25, 50, and 100 μM) for 2 h and then exposed to OGD/R. (a) Viability of SH-SY5Y cells was determined using an MTT assay. (b) LDH activity was measured using an LDH assay kit. (c and d) Cell apoptosis was detected via flow cytometry. (e) Caspase-3 activity was measured using a caspase-3 activity detection kit. (f) Cleaved caspase-3 protein detection was detected using western blotting. (g) Cleaved caspase-3/GAPDH ratio. **p < 0.01 vs Control; ^#,##^ *p* < 0.05, 0.01 vs OGD/R.

SH-SY5Y cells were pre-treated with the indicated concentrations of ART (25, 50, and 100 μM) for 2 h and then exposed to OGD/R. The results of the MTT and LDH release cell death assays indicated that OGD/R-exposure significantly repressed the viability of SH-SY5Y cells (Figure 2a) and enhanced LDH activity (Figure 2b). FCM results showed that OGD/R treatment markedly increased SH-SY5Y cell apoptosis (Figure 2c and d), and caspase-3 activity in OGD/R-injured SH-SY5Y cells was significantly increased (Figure 2e). Western blot analysis revealed that the cleaved caspase-3 protein expression and ratio of cleaved caspase-3/GAPDH were apparently increased in OGD/R-injured SH-SY5Y cells (Figure 2f and g). All the effects could be reversed by ART treatment. This reversal was dependent on ART concentration, and the reversal effect was positively correlated with ART concentration. These results indicated that ART increased the viability and reduced the apoptosis of OGD/R-exposed SH-SY5Y cells in a dose-dependent manner.

**Figure 3:**
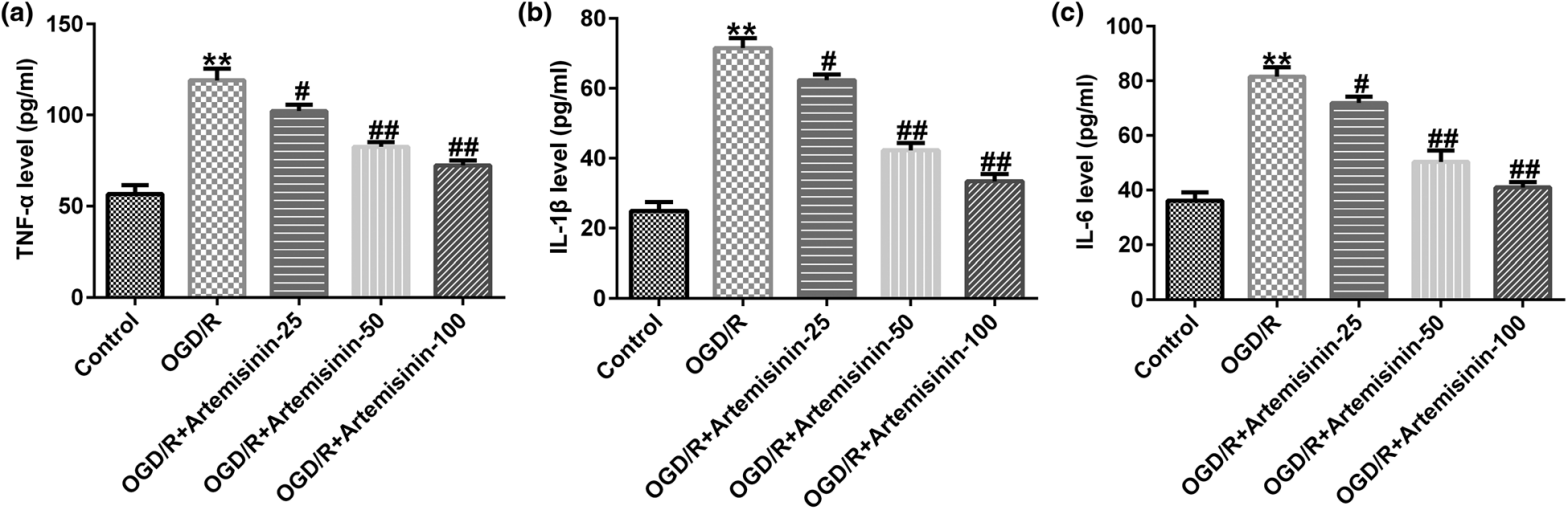
Effects of ART on cell inflammation in hypoxia-glucose deprivation and reoxygenation (OGD/R)-induced SH-SY5Y cells. SH-SY5Y cells were pre-treated with the indicated concentrations of ART (0, 25, 50, and 100 μM) for 2 h and then exposed to OGD/R. (a–c) ELISA was used to determine the secretion of TNF-α, IL-1β, and IL-6 in the supernatant of SH-SY5Y cells. **p < 0.01 vs Control; ^#,##^ *p* < 0.05, 0.01 vs OGD/R.

The secretion of TNF-α, IL-1β, and IL-6 in SH-SY5Y cells was detected using ELISA. The results indicated that the secretion of TNF-α, IL-1β, and IL-6 was increased in OGD/R-exposed SH-SY5Y cells (Figure 3a–c), and ART decreased the secretion of TNF-α, IL-1β, and IL-6 in a dose-dependent manner in OGD/R-exposed SH-SY5Y cells (Figure 3a–c).

**Figure 4:**
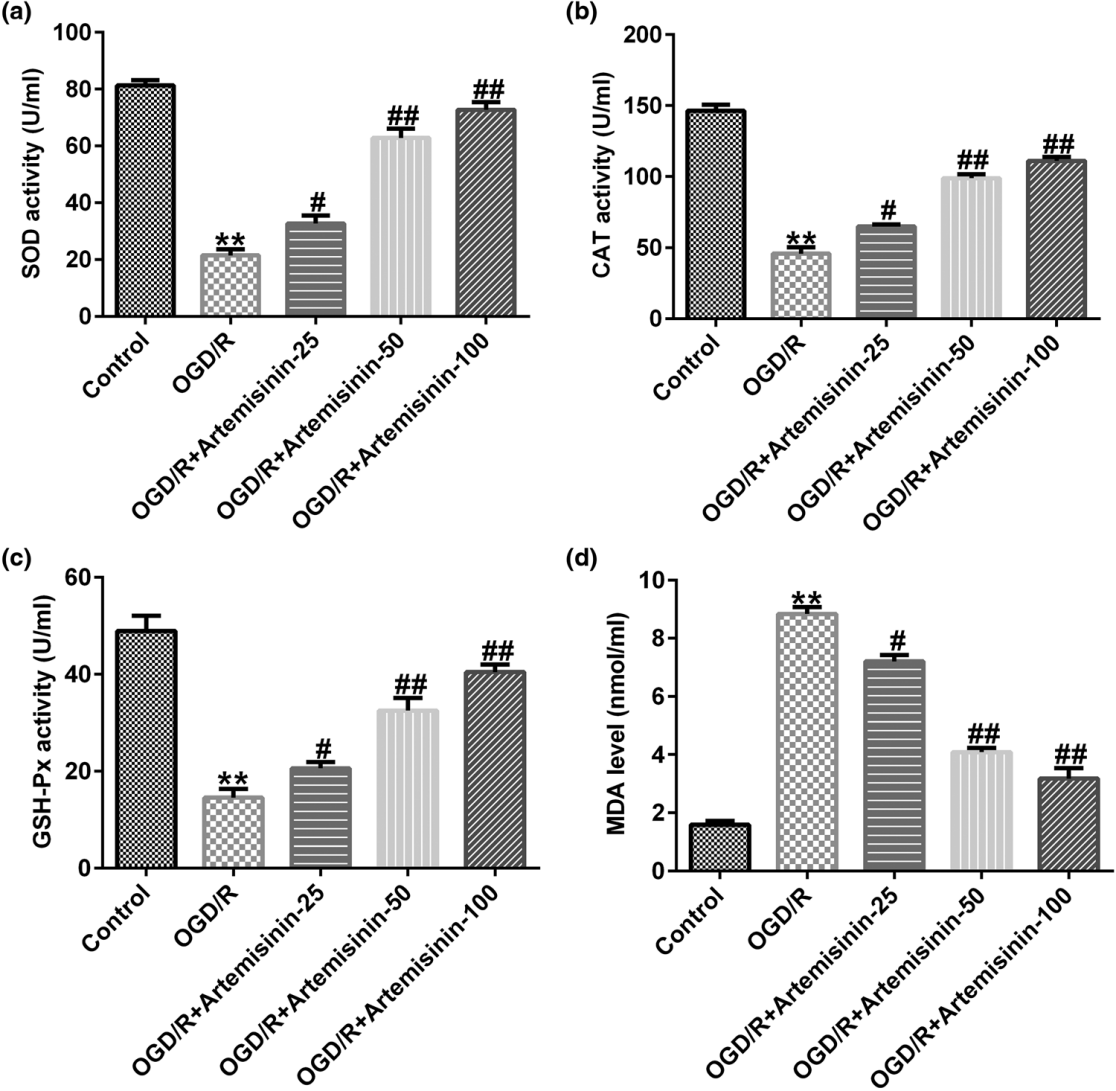
Effects of ART on the oxidative stress in OGD/R-induced SH-SY5Y cells. SH-SY5Y cells were pre-treated with the indicated concentrations of ART (0, 25, 50, and 100 μM) for 2 h and then exposed to OGD/R. Next the activities of SOD (a), CAT (b), and GSH-Px (c) and the level of MAD (d) were determined. **p < 0.01 vs Control; ^#,##^ *p* < 0.05, 0.01 vs OGD/R.

The activities of SOD, CAT, and GSH-Px in SH-SY5Y cells were significantly inhibited, and the level of malondialdehyde (MAD) was significantly increased by OGD/R exposure (Figure 4a–d). In OGD/R-exposed SH-SY5Y cells, ART treatment alleviated the inhibition of SOD, CAT, and GSH-Px activities and increased MAD levels in a dose-dependent manner (Figure 4a–d).

**Figure 5:**
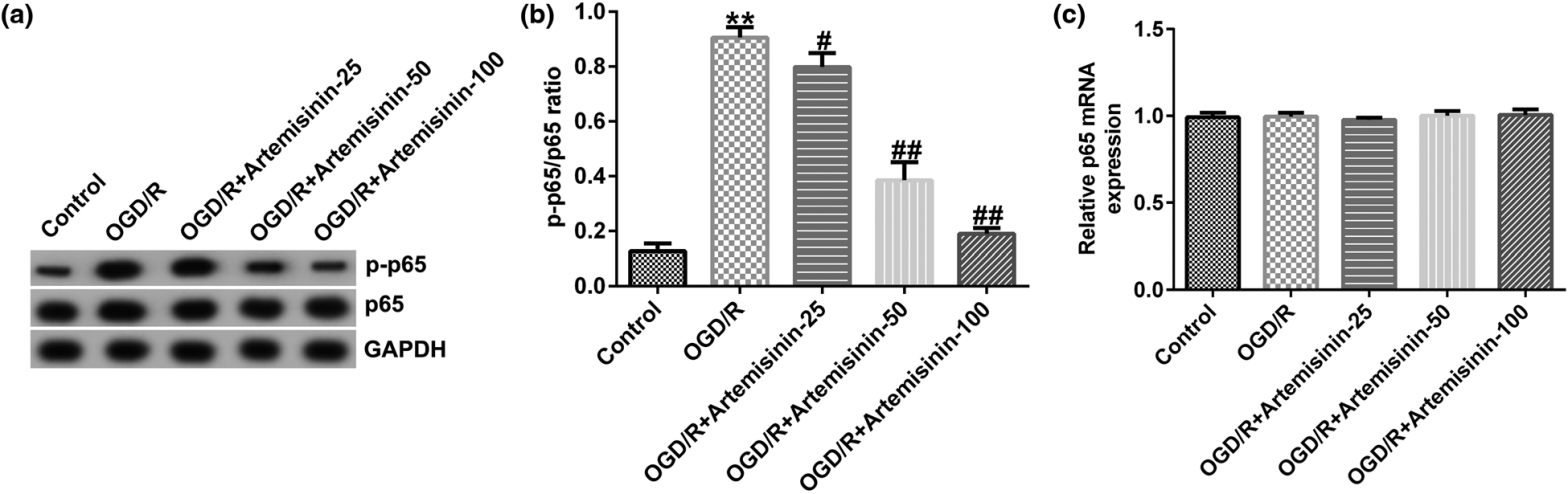
ART inhibits the activation of NF-κB signaling pathway in OGD/R-induced SH-SY5Y cells. SH-SY5Y cells were pre-treated with the indicated concentrations of ART (0, 25, 50, and 100 μM) for 2 h and then exposed to OGD/R. (a) Protein expressions of p-p65 and p65 were measured using western blotting. (b) p-p65/p65 ratio. (c) mRNA expression of p65 was determined via RT-qPCR. **p < 0.01 vs Control; ^#,##^ *p* < 0.05, 0.01 vs OGD/R.

Western blotting was used to assess the expression of p65 and p-p65, and RT-qPCR was performed to analyze the transcription level of p65. The results indicated that p-p65 expression and the ratio of p-p65/p65 were notably increased, and ART treatment reduced p-p65 expression and the ratio of p-p65/p65 in OGD/R-exposed SH-SY5Y cells in a dose-dependent manner (Figure 5a and b). There was no significant change in p65 transcription levels among the groups (Figure 5c).

#### 3.1.2 ART protects SH-SY5Y cells from OGD/R-induced injury by inhibiting the NF-κB signaling pathway

**Figure 6:**
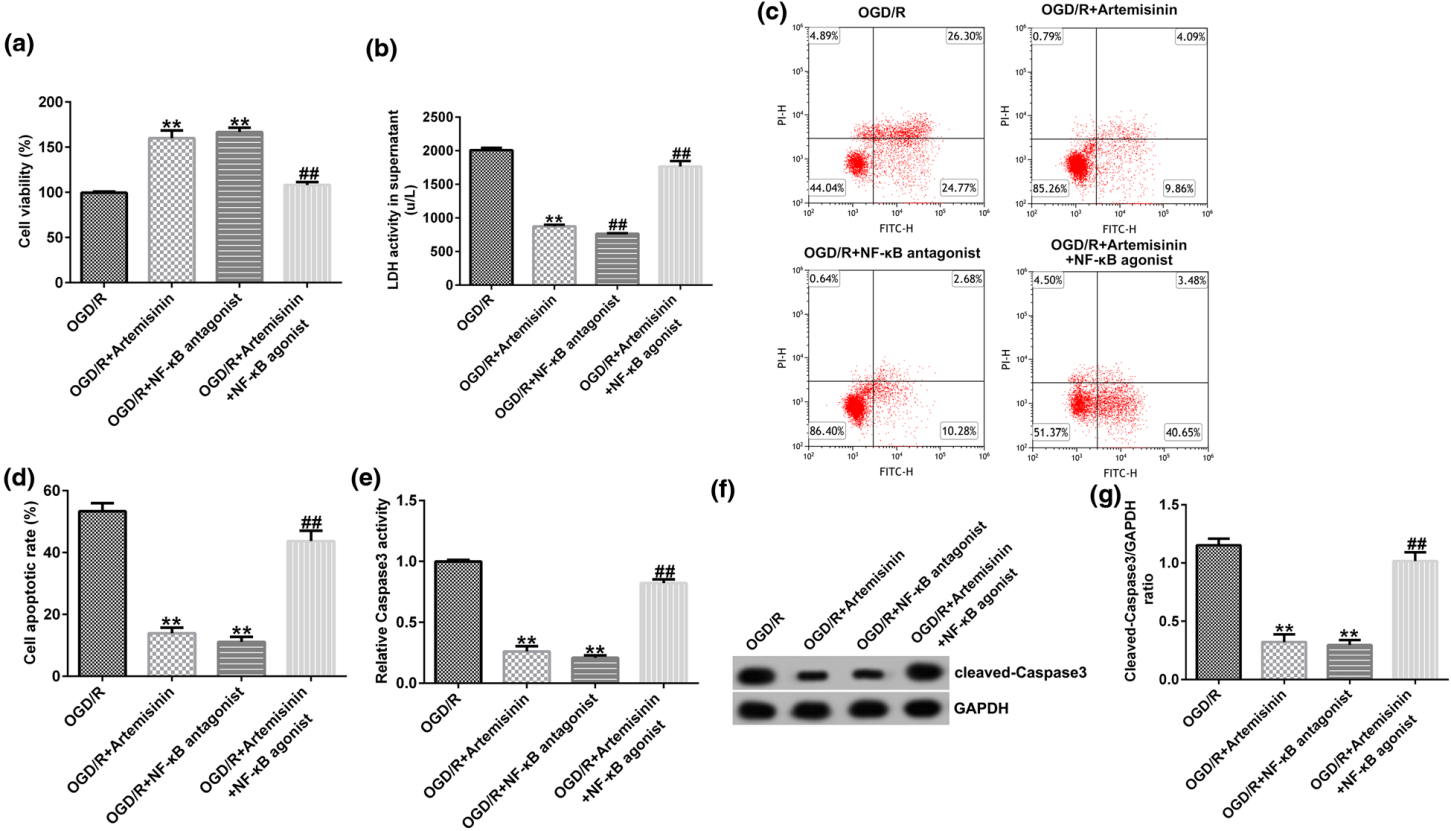
NF-κB agonist reverses the effects of ART on the viability and apoptosis of SH-SY5Y cells triggered by OGD/R exposure. SH-SY5Y cells were pre-treated with 100 μM ART, an NF-κB antagonist, or 100 μM ART + NF-κB agonist for 2 h and then exposed to OGD/R. (a) SH-SY5Y cell viability was determined using an MTT assay. (b) LDH activity was measured using an LDH assay kit. (c and d) Cell apoptosis was detected via flow cytometry (e) Caspase-3 activity was measured using a caspase-3 activity detection kit. (f) Protein expression of cleaved caspase-3 was detected using western blotting. (g) Cleaved caspase-3/GAPDH ratio. **p < 0.01 vs OGD/R; ^##^ *p* < 0.05, 0.01 vs OGD/R + ART.

Findings indicated that OGD/R-exposed SH-SY5Y cell viability was significantly improved and LDH activity was reduced by treatment with ART or an NF-κB agonist (Figure 6a and b). The NF-κB agonist reversed the ART-induced increase in cell viability in OGD/R-exposed SH-SY5Y cells (Figure 6a and b). Apoptosis was distinctly decreased by treatment with ART or an NF-κB agonist, whereas apoptosis was distinctly increased by co-treatment with ART and an NF-κB agonist in OGD/R-exposed SH-SY5Y cells compared with that of ART treatment alone (Figure 6c and d). Caspase-3 activity in OGD/R-exposed SH-SY5Y cells was markedly inhibited by treatment with ART or an NF-κB agonist; however, co-treatment with ART and the NF-κB agonist markedly improved caspase-3 activity in OGD/R-exposed SH-SY5Y cells compared with that of ART treatment alone (Figure 6e). Cleaved caspase-3 protein expression and the ratio of cleaved caspase-3/GAPDH were significantly decreased by treatment with ART or an NF-κB agonist. Compared with that of the ART treatment group, cleaved caspase-3 expression and the ratio of cleaved caspase-3/GAPDH were significantly increased by co-treatment with ART and NF-κB agonist in OGD/R-exposed SH-SY5Y cells (Figure 6f and g).

**Figure 7:**
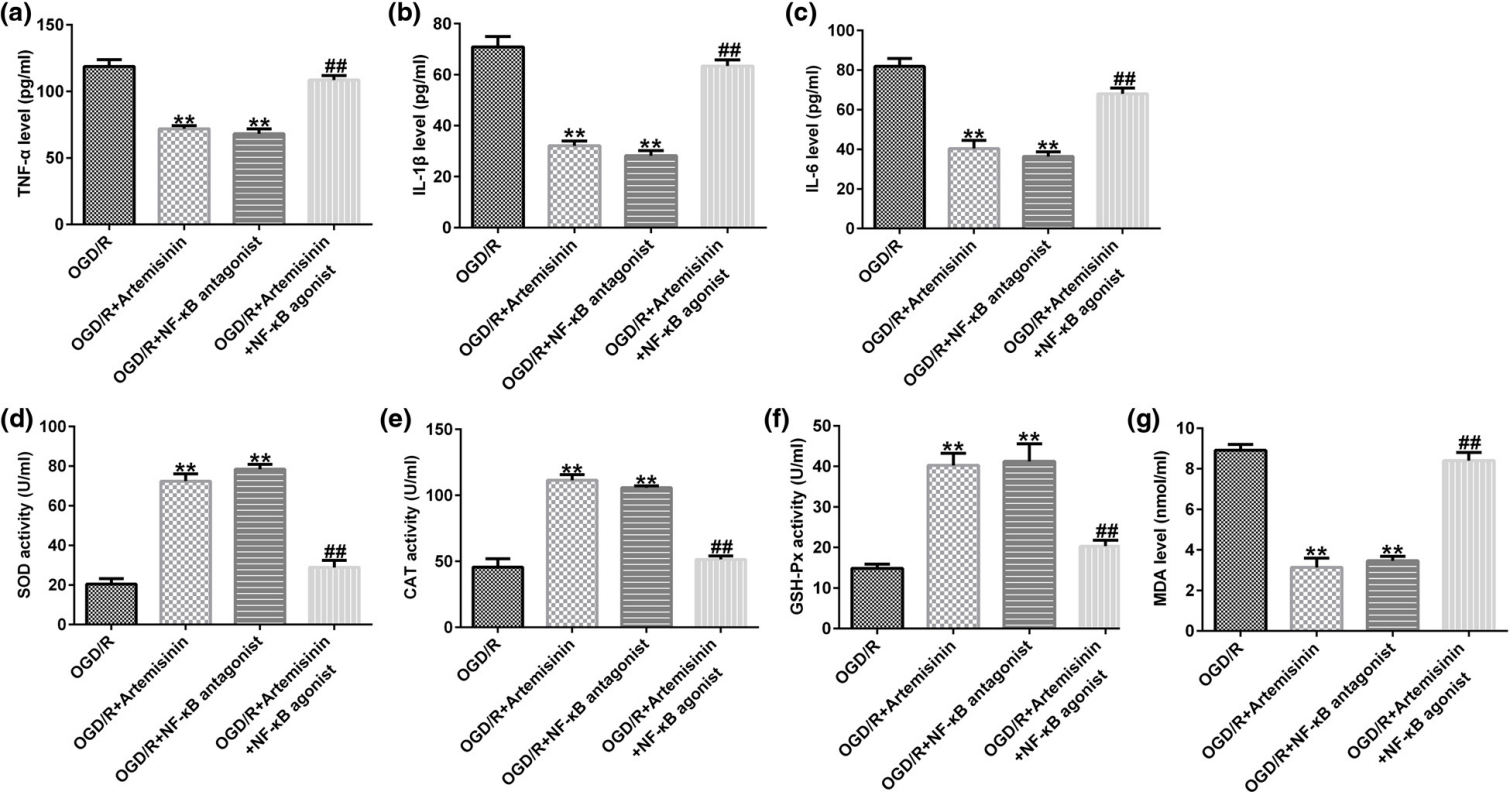
NF-κB agonist reverses the effects of ART on inflammation and oxidative stress of SH-SY5Y cells triggered by OGD/R exposure. SH-SY5Y cells were pre-treated with 100 μM ART, an NF-κB antagonist, or 100 μM ART + NF-κB agonist for 2 h and then exposed to OGD/R. (a–c) ELISA was used to determine the secretion of TNF-α, IL-1β, and IL-6 in the supernatant of SH-SY5Y cells. (d–g) Activities of SOD, CAT, and GSH-Px and the level of MAD were determined. **p < 0.01 vs OGD/R; ^##^ *p* < 0.05, 0.01 vs OGD/R + ART.

The secretion of TNF-α, IL-1β, and IL-6 was evidently decreased in OGD/R-exposed SH-SY5Y cells by treatment with ART or an NF-κB agonist, and co-treatment with ART and the NF-κB agonist evidently increased the secretion of TNF-α, IL-1β, and IL-6 compared with that of ART treatment alone (Figure 7a–c). In OGD/R-exposed SH-SY5Y cells, the activities of SOD, CAT, and GSH-Px were markedly inhibited by treatment with ART or an NF-κB agonist, whereas the inhibitory effect was attenuated by co-treatment with ART and the NF-κB agonist (Figure 7d–f). ART or NF-κB agonist treatment significantly decreased the level of MAD in OGD/R-exposed SH-SY5Y cells; however, the level of MAD was significantly increased by co-treatment with ART or an NF-κB agonist (Figure 7g).

**Figure 8:**
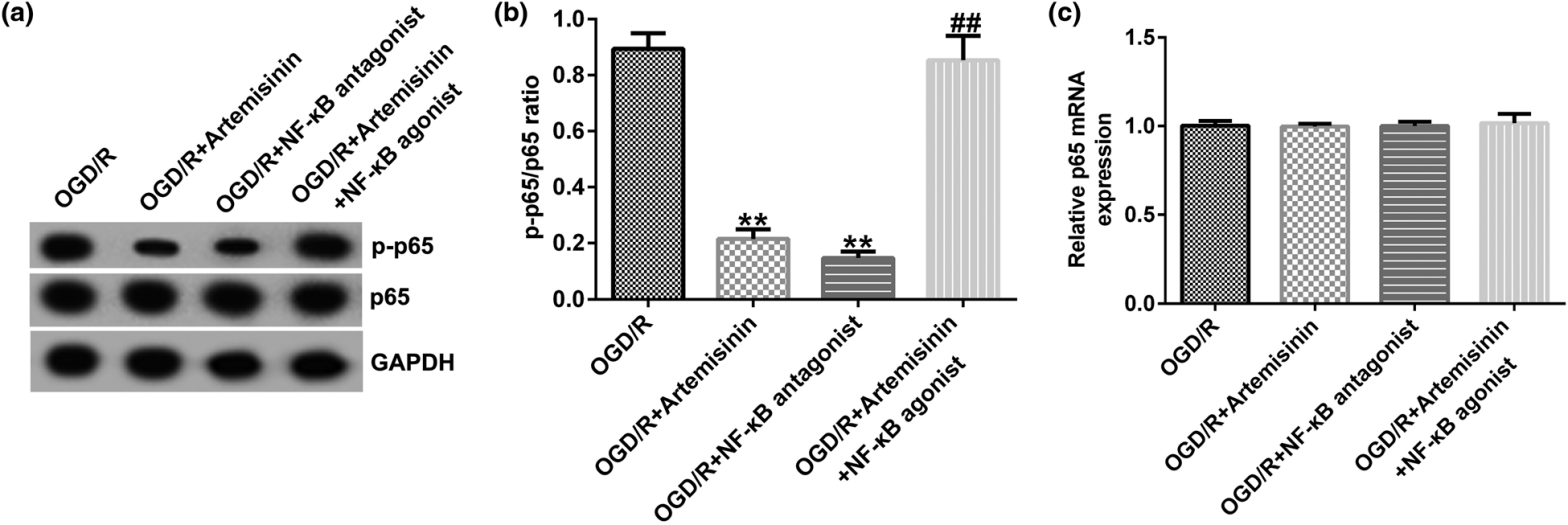
NF-κB agonist reverses the inhibitory effect of ART on the NF-κB signaling pathway in OGD/R-exposed SH-SY5Y cells. SH-SY5Y cells were pre-treated with 100 μM ART, an NF-κB antagonist, or 100 μM ART + NF-κB agonist for 2 h and then exposed to OGD/R. (a) Protein expressions of p-p65 and p65 were measured using western blotting. (b) p-p65/p65 ratio. (c) mRNA expression of p65 was determined via RT-qPCR. **p < 0.01 vs OGD/R; ^##^ *p* < 0.05, 0.01 vs OGD/R + ART.

The findings suggested that p-p65 expression and the ratio of p-p65/p65 were significantly inhibited in OGD/R-injured SH-SY5Y cells after treatment with ART or the NF-κB agonist; however, the inhibitory effect of ART on OGD/R-injured SH-SY5Y cells was reversed by co-treatment with ART and the NF-κB agonist (Figure 8a and b). The transcription level of p65 did not differ among the groups (Figure 8c). ART attenuates OGD/R injury by inhibiting the NF-κB signaling pathway.

## 4 Discussion

Ischemic strokes cause severe disability and death, accounting for approximately 87% of all stroke cases [19,32]. Extensive studies have revealed that strokes cause complex cellular biochemical events that ultimately result in necrosis, apoptosis, or autophagy in ischemic areas [24,25,26]. A comprehensive understanding of neuronal death during ischemic brain injury facilitates the development of new therapies. The pathogenesis of ischemic stroke includes excitatory toxicity, oxidative stress, inflammatory responses, and apoptosis [33]. There is increasing evidence that ART derivatives participate in I/R injury. In this study, we investigated the effects of ART on OGD/R-exposed SH-SY5Y cells. The results indicated that ART increased the viability and decreased the apoptosis of SH-SY5Y cells exposed to OGD/R in a dose-dependent manner.

The inflammatory response caused by leukocyte infiltration after cerebral ischemia plays an important role in the occurrence and development of cerebral I/R injury [34]. The inflammatory cascade after cerebral ischemia is a dynamic process involving the interaction of various cells in the ischemic area, which can cause a second injury after cerebral ischemia. The damaged brain cells produce a large number of platelet activating factors, TNF, IL-6, and other inflammatory mediators after cerebral ischemia [35,36]. The regulatory effect of ART on pro-inflammatory cytokine expression has been widely reported. In LL37-induced rosacea-like mice, ART and its derivatives significantly inhibited the expression of pro-inflammatory factors (IL-1β, IL-6, and TNFα) and TLR2 [37]. In OGD/R-exposed SH-SY5Y cells, our results indicated that the secretion of inflammatory cytokines TNF-α, IL-1β, and IL-6 was significantly increased, and ART treatment inhibited the OGD/R-induced SH-SY5Y cell inflammatory response. The secretion of TNF-α, IL-1β, and IL-6 was inhibited by ART treatment in a dose-dependent manner.

Oxidative stress mediated by ROS is another important factor associated with cerebral I/R injury dysfunction. Antioxidant therapy helps to reduce neuronal variability and improve neurological prognosis [38,39]. Thus, we examined oxidative stress in SH-SY5Y cells following exposure to OGD/R. The levels of free radicals are difficult to measure because of their relatively short half-lives. Therefore, the activities of antioxidant enzymes, such as SOD, CAT, and GSH-Px, and lipid peroxidation by-products, such as MDA, can be used to indirectly evaluate the level of free radicals [40]. In OGD/R-induced SH-SY5Y cells, the antioxidant enzyme (SOD, CAT, and GSH-Px) activities were significantly decreased, and the MAD level was significantly increased. ART contains an endoperoxide bridge, which makes it an antioxidant. The mechanism of killing malaria parasites is related to the production of destructive ROS induced by ART [41]. Our results revealed that ART treatment could alleviate the inhibition of SOD, CAT, and GSH-Px activities and decrease MAD levels induced by OGD/R exposure in a dose-dependent manner.

NF-κB activation is involved in various cellular processes, including the regulation of cell survival, apoptosis, inflammation, and oxidative stress [42,43]. Moreover, NF-κB plays an important role in I/R injury [9,14,16,44]. NF-κB pathway is activated in cerebral I/R [45,46]. After cerebral I/R, NF-κB downregulation can alleviate cerebral edema and neurological dysfunction, and the NF-κB signaling pathway plays an important role in ventricular remodeling after myocardial infarction [16,47,48]. Matsui et al. found that an NF-κB inhibitor could reduce I/R injury in animal experiments [49]. In our study, p-p65 expression and the p-p65/p65 ratio in OGD/R-exposed SH SY5Y cells were significantly increased, and ART treatment could reduce p-p65 expression and the p-p65/p65 ratio in a dose-dependent manner. NF-κB agonist treatment could significantly increase the cell viability, decrease the apoptosis, and inhibit the inflammatory response and oxidative stress of OGD/R-exposed SH-SY5Y cells, suggesting that NF-κB is involved in the regulation of OGD/R-induced cell proliferation, apoptosis, inflammation, and oxidative stress. The NF-κB agonist and ART have similar effects on OGD/R-exposed SH-SY5Y cells. We then investigated whether the regulation of ART in OGD/R-exposed SH SY5Y cells is related to NF-κB pathway. As expected, all the effects of ART on OGD/R-exposed SH SY5Y cells could be reversed by co-treatment with an NF-κB agonist and ART.

Overall, ART protects neurons from OGD/R-induced damage *in vitro* by inhibiting the NF-κB signaling pathway. However, to make the effect of ART in cerebral ischemia and reperfusion injury more convincing, *in vivo* investigations should be performed. This study did not study the effect of ART in cerebral ischemia and reperfusion injury in animal models, and this was a limitation of the present study. We will delve into this in our next study.

## 5 Conclusion

We found that ART can significantly promote cell viability and inhibit cell apoptosis, inflammatory response, and oxidative stress in OGD/R induced SH SY5Y cells. The effect of ART on OGD/R-exposed SH SY5Y cells is achieved by inhibiting the NF-κB signaling pathway. These results indicate that ART may be a potential agent for the treatment of cerebral I/R injury.
